# Protection following BNT162b2 booster in adolescents substantially exceeds that of a fresh 2-dose vaccine

**DOI:** 10.1038/s41467-022-29578-w

**Published:** 2022-04-13

**Authors:** Ofra Amir, Yair Goldberg, Micha Mandel, Yinon M. Bar-On, Omri Bodenheimer, Nachman Ash, Sharon Alroy-Preis, Amit Huppert, Ron Milo

**Affiliations:** 1 Technion—Israel Institute of Technology, Haifa, Israel; 2 The Hebrew University of Jerusalem, Jerusalem, Israel; 3 Department of Plant and Environmental Sciences, Weizmann Institute of Science, Rehovot, Israel; 4 Israel Ministry of Health, Jerusalem, Israel; 5 The Bio-statistical and Bio-mathematical Unit, The Gertner Institute for Epidemiology & Health Policy Research, Sheba Medical Center, Ramat Gan, Israel; 6 The Sackler Faculty of Medicine, Tel Aviv University, Tel Aviv, Israel

**Keywords:** Public health, Epidemiology, SARS-CoV-2

## Abstract

Israel began administering a BNT162b2 booster dose to restore protection following the waning of the 2-dose vaccine. Biological studies have shown that a “fresh” booster dose leads to increased antibody levels compared to a fresh 2-dose vaccine, which may suggest increased effectiveness. To compare the real-world effectiveness of a fresh (up to 60 days) booster dose with that of a fresh 2-dose vaccine, we took advantage of a quasi-experimental study that compares populations that were eligible to receive the vaccine at different times due to age-dependent policies. Specifically, we compared the confirmed infection rates in adolescents aged 12–14 (215,653 individuals) who received the 2-dose vaccine and in adolescents aged 16–18 (103,454 individuals) who received the booster dose. Our analysis shows that the confirmed infection rate was lower by a factor of 3.7 (95% CI: 2.7 to 5.2) in the booster group.

## Abstract (web summary)

This study compares SARS-CoV-2 infection rates following a recent second or third dose of the BNT162b2 (Pfizer/BioNTech) vaccine. They use data from Israel for 12–14 year olds (second dose) and 16–18 year olds (third dose), and find a 3.7-fold higher risk in the second-dose group.

## Introduction

Laboratory studies showed that a BNT162b2 booster dose significantly increases the antibody neutralization and the IgG titers levels compared to a second dose^1,2^, which suggests possible increased protection against infection^3,4^. In this study, we sought to compare the real-world effectiveness of a “fresh” (up to 60 days) BNT162b2 booster dose in preventing infection to that of a “fresh” two-dose BNT162b2 vaccine. Population studies have shown that the booster is highly effective in restoring protection against infection, reducing the rate of confirmed infections and severe outcomes by several folds compared to doubly-vaccinated individuals 5 months after vaccination^5–7^. Assessing whether and to what extent a booster dose increases protection compared to “fresh” two doses is challenging due to selection bias. Individuals who received the booster dose chose to vaccinate earlier, which has been shown to be correlated with important risk and exposure factors such as high sociodemographic status^8^.

To mitigate this bias, we use a quasi-experimental study^9^. In Israel (upon the United State’s Food and Drug Administration approval), residents aged 16 or older were eligible to vaccinate starting February 2021, while teenagers aged 12–15 became eligible to receive the vaccine only in June 2021. Following observations of waning in the protection conferred by the BNT162b2 (Pfizer-BioNTech) vaccine^10^, Israel began the administration of a third (BNT162b2 booster) dose on July 30, 2021 to individuals 60 years or older. Starting August 29, 2021, every person aged 16 or older who received the second dose at least 5 months earlier was eligible for a booster dose. We utilize the age-dependent vaccination policy to compare the protection conferred by a fresh two-dose vaccine to that conferred by a fresh booster dose.

Our main analysis compared the rates of confirmed infections between September 12, 2021 and October 9, 2021 (fourth wave in Israel, which was Delta-dominant) in two cohorts: individuals aged 16–18 who received the booster dose and individuals aged 12–14 who were recently vaccinated (2 doses). Individuals in both cohorts chose to vaccinate soon after becoming eligible. For the doubly-vaccinated cohort, we only included persons who were vaccinated for less than 60 days to avoid the effect of waning immunity^10^. We used a Poisson regression (see Methods) to estimate the confirmed infection rates in the two cohorts during the 4-week study period. To compare the protection of the fresh booster to that of fresh two doses, we then calculated the rate ratio between confirmed infection rates in the two cohorts. We did not analyze protection against severe disease as there were only a few cases, as expected for such young ages.

The quasi-experimental study is not bias-free as a controlled experiment, as prior to the vaccination campaign, the younger age group had a somewhat lower rate of confirmed infections than the 16–18 group, which is in line with prior studies showing lower infectivity and susceptibility in younger children^11^. On the other hand, following the vaccination campaign, the older age group might have higher indirect protection from being in an environment with higher vaccination rates. Since there are several possible biases resulting from the different ages in the two cohorts, we also report several sensitivity analyses, as well as a secondary analysis comparing the protection of the cohorts to unvaccinated individuals in corresponding age groups.

## Results

**Fig. 1:**
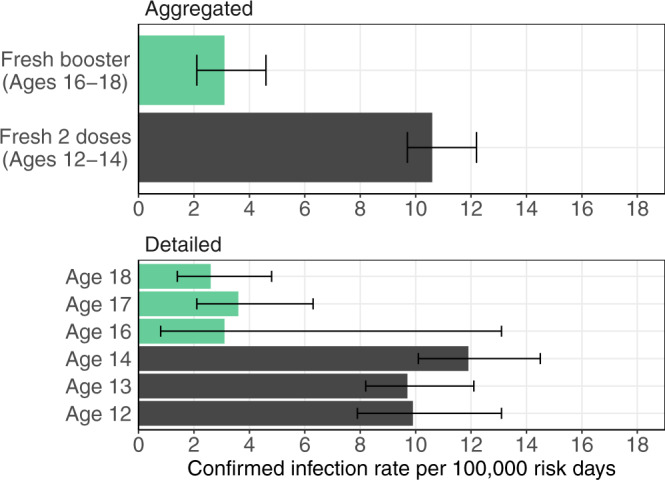
Estimated covariate-adjusted rates of confirmed infections per 100,000 at-risk days. Estimates were obtained from Poisson regression analysis for the study period from September 12, 2021 to October 9, 2021, stratified by cohorts, with *n* = 4,361,550 days at risk. The top plot shows the results of the main analysis of the two study cohorts. The bottom plot shows covariate-adjusted rates of confirmed infections for each age group. Errors bars show 95% confidence intervals (not adjusted for multiplicity).

The main analysis shows that the confirmed infection rate in the fresh booster cohort was 3.7-fold (95% CI: 2.7–5.2) lower than in the fresh two-dose vaccine cohort (Fig. 1). The infection rate in the booster cohort was 3.3 (95% CI: 2.4–4.6) per 100,000 at-risk days, compared to 12.4 (95% CI: 11.4–14) in the doubly-vaccinated cohort.

To test the robustness of the results the following sensitivity analyses were conducted. First, the 17–18 age group comprises individuals who graduated from high school prior to the study period. Therefore, they may differ from the other age groups that are exposed to other students. Indeed, we observed lower infection rates in the 17–18-year-old unvaccinated age group compared to younger age groups (Fig. S2). We, therefore, repeated the analysis after removing this age group and found a similar rate ratio of confirmed infections, with a rate lower by a factor of 3.5 (95% CI: 2.0, 6.1) in the fresh booster cohort compared to the two-dose cohort. We further restricted the analysis to a comparison between the 14-year-old doubly-vaccinated individuals and the 16-year-old boosted individuals, which are the closest age groups in our natural experiment; The rate ratio increased to a factor of 4.6 (95% CI: 2.5, 8.1).

Second, because booster uptake rates were higher in the General Jewish population, we conducted an additional analysis that only included this sub-population. This analysis showed that the rate of confirmed infection was lower by a factor of 4.1 (95% CI: 2.8, 6.2) in the fresh booster cohort. Lastly, we analyzed confirmed infection rates over a longer study period—from September 12 to Oct 24, resulting in an estimate of a 3.5-fold (95% CI: 2.4, 5.2) reduction in confirmed infection rates for the booster cohort. This analysis was based on data on more individuals and confirmed infection events, but also included individuals who were doubly-vaccinated later. Overall, all sensitivity analyses yielded similar results, with confirmed infection rates in the fresh booster cohort lower by a factor of 3–5 compared to the rates in the fresh two-dose cohort.

In the primary quasi-experimental analysis, biases may occur due to age differences, either because natural protection is correlated with age^11^, or due to behavioral differences. Our secondary analysis compared the rates of confirmed infections in three cohorts: individuals aged 16–18 who received the booster dose, individuals aged 12–14 who were recently vaccinated (two doses), and a new cohort of individuals aged 16–18 who were recently vaccinated with two doses. The 12–14 vaccinated group is similar to the booster group in that individuals chose to vaccinate soon after becoming eligible. However, due to the age difference, this group also seems to be more exposed than the 16–18 group according to infections among unvaccinated individuals (see Fig. S2). The 16–18 doubly-vaccinated unboosted cohort presumably has a similar exposure risk as that of the 16–18 unvaccinated cohort and does not suffer from this bias, but could introduce a behavioral bias as they were vaccinated relatively late. In this analysis, we first compared the rate ratios of confirmed infections between the vaccinated groups (either a fresh booster or a fresh 2-dose vaccine) and the unvaccinated groups in the corresponding age groups (e.g., the rate ratio of confirmed infection in the booster cohort relative to the 16–18 unvaccinated group). We then compared the rate ratio observed in the fresh booster cohort to the rate ratio observed in each of the fresh two-dose vaccine cohorts.

The secondary analysis results are summarized in Fig. S3. The rate ratio comparing the fresh booster cohort with the unvaccinated cohort was higher than the rate ratios observed in the fresh 2-dose vaccine groups by a factor of 2–3. Compared to unvaccinated individuals 16–18 years old, the confirmed infection rate in the booster cohort was lower by a factor of 26.3 (95% CI: 19.2, 36), while in the two-dose group the rate was lower by a factor of 9.8-fold (95% CI: 5, 16) compared to the unvaccinated individuals (which is 2.7-fold lower than that of the booster group). In the doubly-vaccinated 12–14 cohort, the confirmed infection rate was 12.5-fold (95% CI: 11.2, 13.8) lower compared to the unvaccinated group of that age, which is 2.1-fold lower than the 26.3-fold reduction in confirmed infection rate observed in the booster cohort.

## Discussion

Our analysis shows that a fresh booster dose of the BNT162b2 mRNA vaccine provides improved protection against confirmed infection from the Delta variant compared to fresh two doses of the same vaccine. These results are in line with lab-based findings showing increased antibody responses both in the IgG titers as well as neutralizing antibody levels of the booster dose^1^. Moreover, the neutralizing antibodies after the booster were superior in neutralization assay to antibodies after a second dose against both Delta and Omicron^12–14^. Together, these findings offer a quantitative basis for the inclusion of a booster dose as part of the BNT162b2 regimen.

The study has several limitations. We observe different confirmed infection rates for unvaccinated individuals in different age groups (see Fig. S2) with higher rates up to age 16 and lower rates from age 17 and above. These differences are possibly related to the 17–18 age group graduating from school prior to the study period, which could affect their exposure and testing rates but could also relate to different characteristics of the unvaccinated population at different ages. To account for this bias, we adjusted for age by year in the regression. In the secondary analysis, we also included the doubly-vaccinated unboosted 16–18 cohort which is similar to the booster cohort with respect to age. However, this group chose to vaccinate later, as opposed to the booster cohort which consisted of individuals who were early to vaccinate. This could reflect differences between the populations as shown in prior studies^8^.

In addition to differences in exposure between age groups, there are also possible differences in testing rates. To account for this, we examined the number of Polymerase Chain Reaction (PCR) tests performed by individuals in the different cohorts (see Fig. S4). We found that the vaccinated 12–14 cohort and the booster cohort had similar testing rates. Related to the secondary analysis which also included the vaccinated 16–18 cohort, we observed a somewhat lower testing rate in this group, suggesting that their protection might be overestimated. We also observed that the unvaccinated population in the 12–14 age group (used as a reference in the secondary analysis) were tested at a higher rate than the vaccinated 12–14 cohort and also at a higher rate than the unvaccinated 16–18 age group. Since the protection rates used the unvaccinated groups as a reference, it is possible that the protection of the vaccinated 12–14 group was overestimated in the secondary analysis. Therefore, the increase in the protection conferred by a booster dose compared to two doses might be even higher than estimated in this analysis.

Another limitation of the study is that individuals in the booster cohort chose to vaccinate early (similar to the 12–14 vaccinated cohort) and also chose to receive the booster dose, leading to a potential selection bias in the booster population. In particular, the General Jewish population had a higher uptake rate of the booster dose. The sensitivity analysis that was restricted to this sector revealed a similar 3–6-fold reduction in confirmed infection rates. Similar results were obtained in the sensitivity analysis that extended the study period by two weeks and included a higher number of individuals who received the booster dose.

Lastly, we note that our estimates for protection reflect the real-world effectiveness of the vaccine and likely include indirect effects such as the added protection due to others being vaccinated, and not only the biological protection conferred by the vaccine. Nevertheless, all the sensitivity analyses we conducted showed that protection against confirmed infection after receiving the booster dose was substantially higher than that after two doses, suggesting that the booster dose indeed improves protection beyond that of only two doses.

## Methods

### Ethics

The study was approved by the Institutional Review Board of the Sheba Medical Center. Helsinki approval number: SMC-8228-21. The investigators did not have access to de-anonymized information.

### Description of the data

The analysis is based on the Israel Ministry of Health’s database, as described in our previous studies^15^. Israel has experienced four pandemic waves, with the Delta (B.1.617.2) variant being the predominant variant during the fourth wave. During the third wave, Israel initiated a very rapid vaccination campaign administering the BNT162b2 vaccine to all adult residents. The campaign was opened on December 20, 2020, initially to people aged 60 years or older, and was then gradually extended until, on February 4, 2021, all individuals aged 16 or older were eligible to receive two doses of the vaccine. After the arrival of the Delta variant to Israel, a new Covid-19 wave began in mid-June 2021. Consequently, on July 30, 2021, the administration of a third (booster) dose was approved, first for people aged 60 years or older, and later for younger age groups^5^.

Israel has a centralized health system, where each resident belongs to one of four health maintenance organizations (HMOs). Polymerase Chain Reaction (PCR) tests for SARS-CoV-2 infections and vaccination against the virus are directly reported to the Ministry of Health (MoH). The MoH established a centralized Covid-19 national database containing regularly updated information on all PCR tests and results, vaccination dates, and follow-up data on all infected individuals. The MoH database also includes basic demographic information on sex, age, place of residency, and population sector. Demographic variables such as age, sex, and sector (general Jewish, Arab, or ultra-Orthodox Jewish) as determined by the individual’s statistical area of residence.

### Study design and population

We used a quasi-experimental design, utilizing the changes made to the vaccine eligibility age cutoffs to estimate the effectiveness of a booster dose to that of a “fresh” two-dose vaccine. The study population included persons who were between the ages of 12–14 or 16–18 starting January 1st, had no documented positive PCR result prior to the study period, had not stayed abroad during the whole study period, and had not been vaccinated with a vaccine different from BNT162b2 before the beginning of the study period. We did not include the 15-year-old group since the data included the age of individuals in one-year groups (based on their age on January 1st, 2021), and the 15-year-old group thus included individuals who were eligible to vaccinate at different times. Table 1 summarizes the characteristics of the study population.

**Table 1:** Demographic and clinical characteristics of the study groups.

Group	Unvaccinated 12–14 Person-days at risk = 2,837,195 *N* (individuals) = 116,841 Unadjusted confirmed infection rate per 100,000 risk days = 191.5		Unvaccinated 16–18 Person-days at risk = 1,818,163 *N* (individuals) = 70,448 Unadjusted confirmed infection rate per 100,000 risk days = 95.5		Vaccinated 12–14 Person-days at risk = 2,645,121 *N* (individuals) = 215,653 Unadjusted confirmed infection rate per 100,000 risk days = 14.8		Vaccinated 16–18 Person-days at risk = 133,613 *N* (individuals) = 67,191 Unadjusted confirmed infection rate per 100,000 risk days = 12.0		Booster 16–18 Person-days at risk = 1,716,429 *N* (individuals) = 103,454 Unadjusted confirmed infection rate per 100,000 risk days = 2.3	
	% person days at risk	# infections	% person days at risk	# infections	% person days at risk	# infections	% person days at risk	# infections	% person days at risk	# infections
Female	51.90%	2,805	53.30%	1,038	51.90%	245	47.40%	7	49.30%	21
Male	48.10%	2,629	46.70%	698	48.10%	146	52.60%	9	50.70%	19
General Jewish	60.50%	2,622	59.90%	930	76.60%	260	53.30%	11	88.20%	29
Arab	15.30%	635	23.00%	303	7.60%	33	8.90%	0	5.40%	4
Ultra-Orthodox	24.10%	2,177	17.20%	503	15.70%	98	37.80%	5	6.40%	7

The booster group comprises individuals of ages 16–18, 14 or more days after they received the booster dose. The doubly-vaccinated groups comprise of individuals 14–60 days after they received the second dose, in ages 12–14 or 16–18. The unvaccinated groups (reference groups in the secondary analysis) comprise of individuals in ages 12–14 or 16–18 who were not vaccinated. Only person-days and events that were used in the main and secondary analyses are presented. The table presents the proportion of person-days at risk instead of the proportion of individuals. Values are presented for the study period— September 12, 2021 to October 9, 2021.

In the primary analysis, the study population was divided into two cohorts. The first cohort included individuals 16–18 years old who recently received the booster dose, and the second cohort included individuals aged 12–14 who were doubly-vaccinated for less than 60 days (to avoid waning effects). To estimate protection conferred by the two-dose and the booster vaccines, we compared their infection rates during the 4-week study period: September 12, 2021 and October 9, 2021 (fourth wave in Israel, which was Delta-dominant).

In a secondary analysis, we also included a third cohort consisting of individuals aged 16–18 who were doubly-vaccinated for less than 60 days. In this analysis, we used unvaccinated individuals as a reference group and estimated the rate ratio of confirmed infection in each cohort relative to the corresponding unvaccinated age group during the study period.

### Statistical analysis

We analyzed the data using a methodology similar to that used in our previous studies^5,6^. The number of confirmed infections and the number of days at risk during the study period were counted for each cohort. A Poisson regression model was fitted adjusting for age (by year), sex, sector (General Jewish, Arab, ultra-Orthodox Jewish), calendar week, and an exposure risk measure. The latter was calculated for each person on each follow-up day according to the proportion of new confirmed infections during the past seven days in their area of residence; this continuous measure was divided into ten categories according to deciles (see Bar-On et al.^5^ for details). An average risk was imputed to individuals with missing data on residency.

### Reporting summary

Further information on research design is available in the Nature Research Reporting Summary linked to this article.

## Supplementary information

Supplementary Information

Reporting Summary

## Data Availability

The individual-level data used in this study cannot be publicly shared even if anonymized due to privacy restrictions.

## Code availability

Code will be shared upon request.
