# Supplementary material for: Protection following BNT162b2 booster in adolescents substantially exceeds that of a fresh 2-dose vaccine
Source: Nat Commun. 2022 Apr 13;13:1971. doi: 10.1038/s41467-022-29578-w (PMC9008037; doi:10.1038/s41467-022-29578-w)

Protection following BNT162b2 booster in adolescents substantially exceeds that of a fresh 2-dose vaccine

Ofra Amir[†1], Yair Goldberg[†1*], Micha Mandel[2], Yinon M. Bar-On[3], Omri Bodenheimer[4], Nachman Ash[4], Sharon Alroy-Preis[4], Amit Huppert[†5,6], Ron Milo[†3]

[1] Technion - Israel Institute of Technology, Israel
[2] The Hebrew University of Jerusalem, Israel
[3]Department of Plant and Environmental Sciences, Weizmann Institute of Science, Israel
[4] Israel Ministry of Health, Israel
[5] The Bio-statistical and Bio-mathematical Unit, The Gertner Institute for Epidemiology & Health Policy Research, Sheba Medical Center, Israel
[6] The Sackler Faculty of Medicine, Tel Aviv University, Israel

[†]These authors contributed equally
*corresponding author
email: yairgo@technion.ac.il

Supplementary Information

Contents

Figure S1 - Study population 3
Figure S2 - Confirmed infection rates in different age groups 4
Figure S3 - Protection compared to unvaccinated individuals 5
Figure S4 - PCR testing rates in different age groups 6

Figure S1 - Study population

The study population included persons who were between the ages of 12-14 or 16-18, had no documented positive PCR result prior to the study period, had not stayed abroad during the whole study period, and had not been vaccinated with a vaccine different from BNT162b2 before the beginning of the study period.

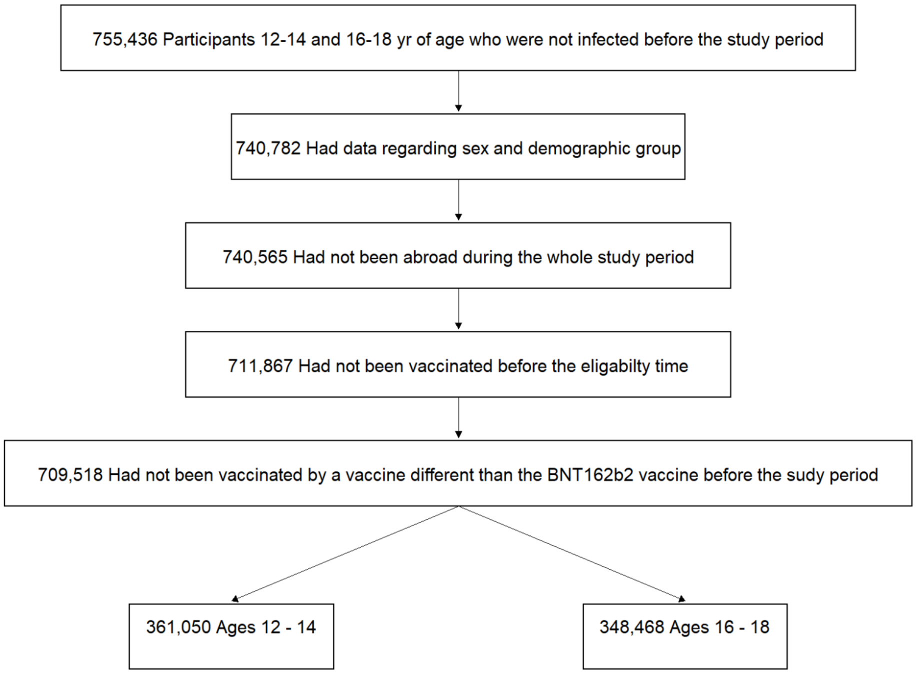

Figure S2 - Confirmed infection rates in different age groups

Crude infection rates per 100,000 at-risk days during the study period between September 12, 2021 to October 9, 2021, stratified by cohort and age. The 15-year-old age group is not shown since it includes individuals who were eligible to vaccinate at different times. Error bars show 95% confidence intervals.

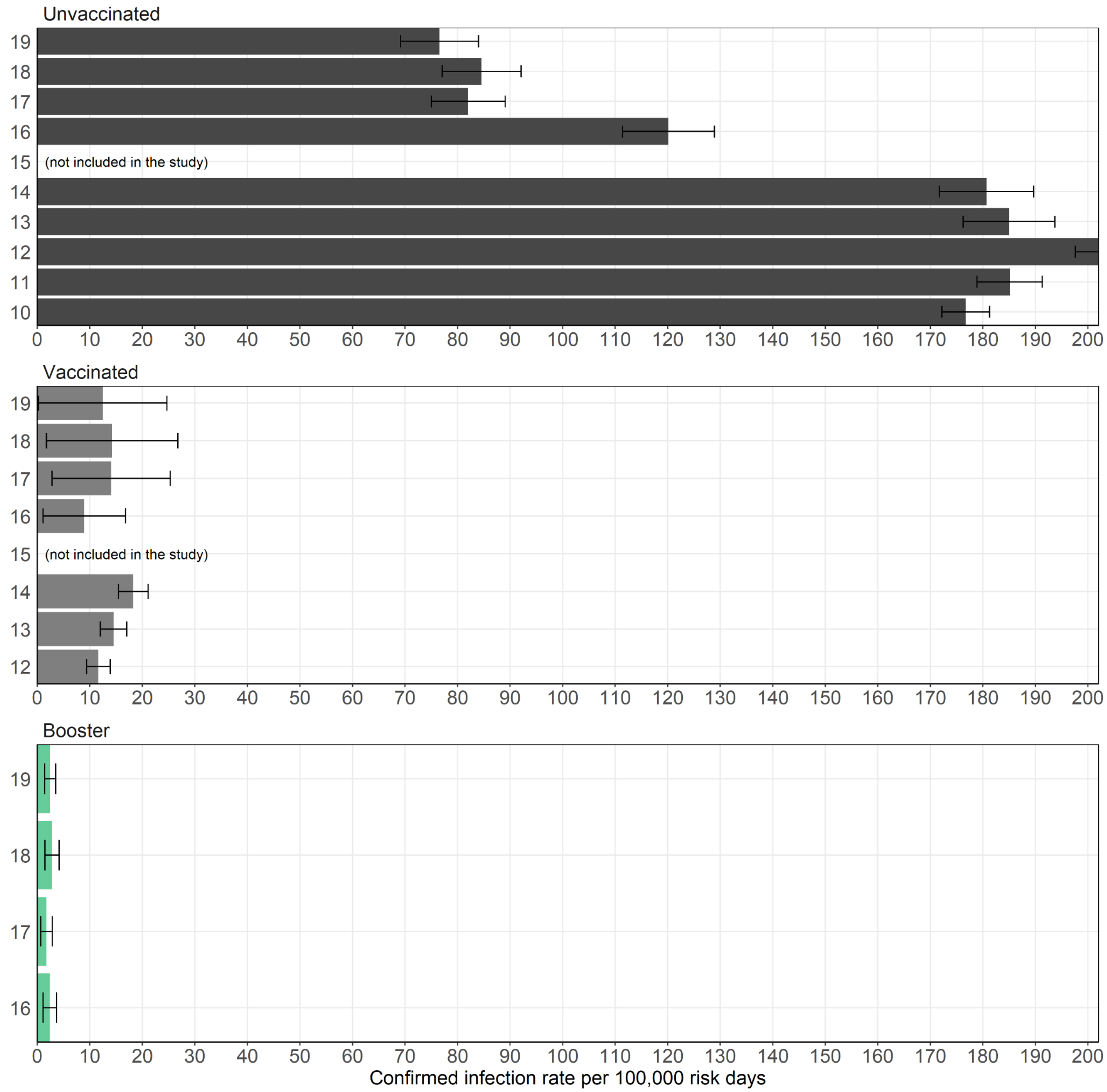

Figure S3 - Protection compared to unvaccinated individuals

Estimated covariate-adjusted rates of confirmed infections per 100,000 at-risk days, based on n=9,150,521 days at risk (A), and factor reductions in confirmed infections compared to unvaccinated individuals in the same age group (B). Obtained from a Poisson regression analysis for the study period September 12, 2021, to October 9, 2021, stratified by cohorts. Error bars show 95% confidence intervals (not adjusted for multiplicity).

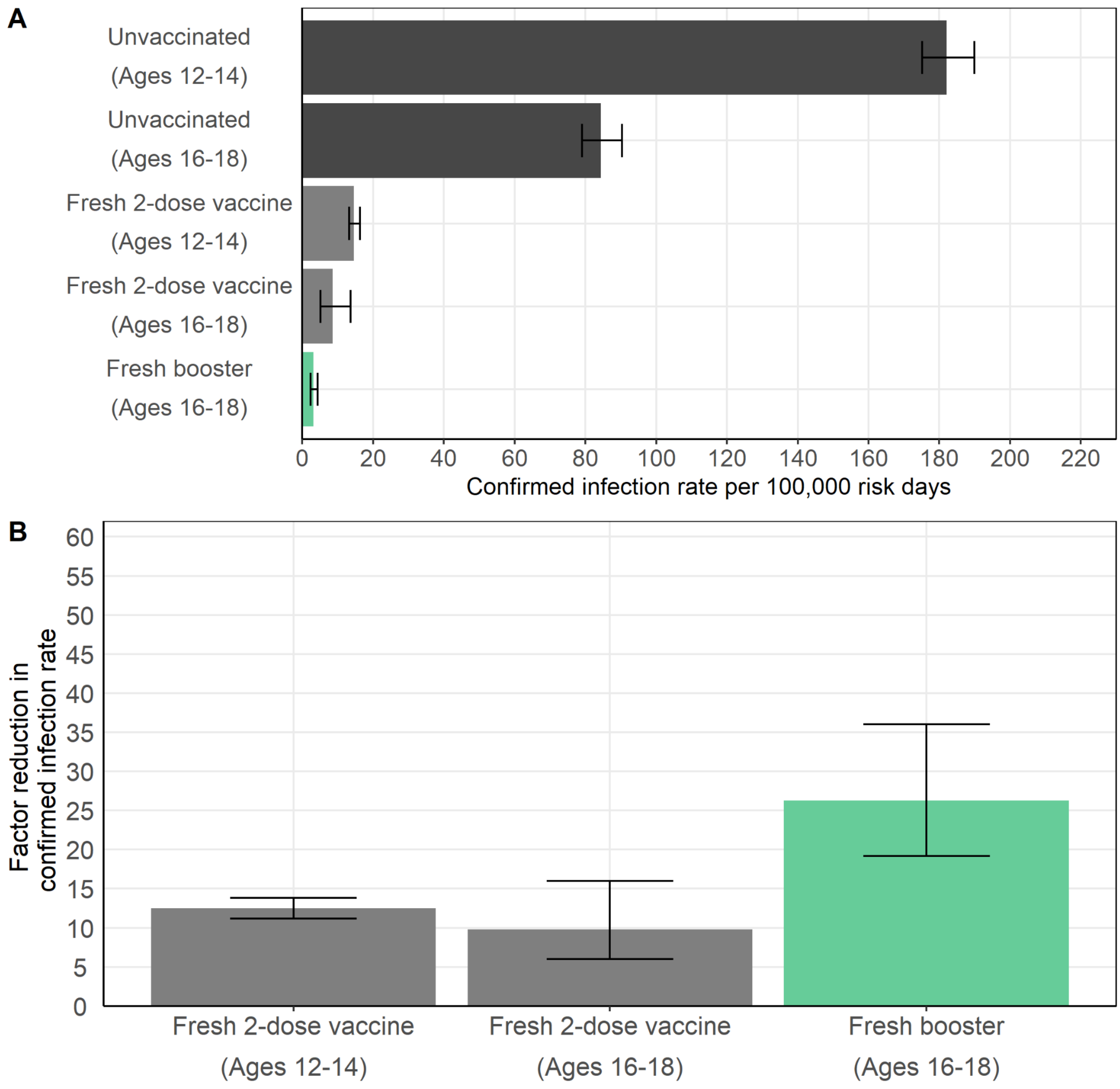

Figure S4 - PCR testing rates in different age groups

PCR test rates per 100,000 risk days during September 2021, stratified by cohorts and ages, based on n=9,150,521 days at risk*. The 15-year-old age group is not shown since it includes individuals who were eligible to vaccinate at different times. Error bars show 95% confidence intervals.*

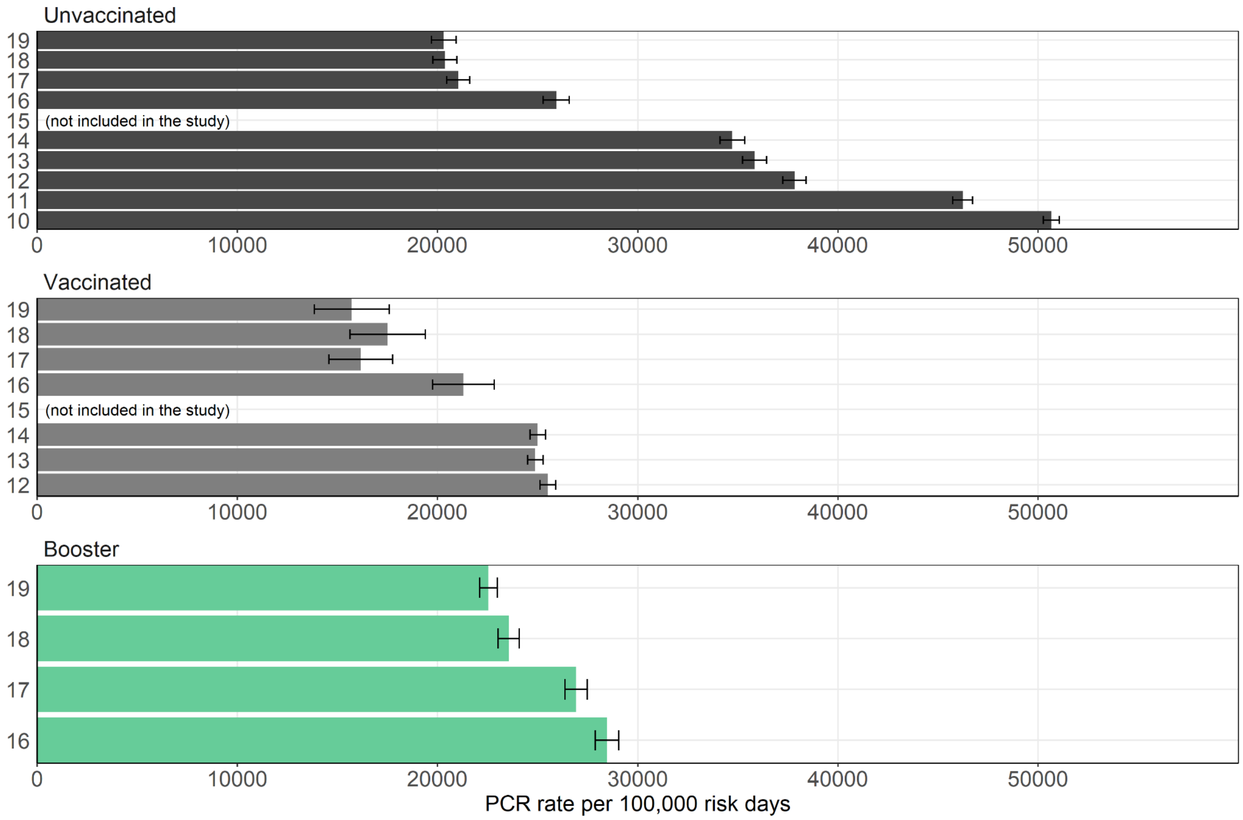

Supplement: Supplementary file 1 — Supplementary Information [file 41467_2022_29578_MOESM1_ESM.pdf]
