# Supplementary material for: Protection following BNT162b2 booster in adolescents substantially exceeds that of a fresh 2-dose vaccine
Source: Nat Commun. 2022 Apr 13;13:1971. doi: 10.1038/s41467-022-29578-w (PMC9008037; doi:10.1038/s41467-022-29578-w)
Supplement: Supplementary file 2 — Reporting Summary [file 41467_2022_29578_MOESM2_ESM.pdf]

nature portfolio

Corresponding author(s): Prof. Yair Goldberg

Last updated by author(s): Mar 13, 2022

Reporting Summary

Nature Portfolio wishes to improve the reproducibility of the work that we publish. This form provides structure for consistency and transparency in reporting. For further information on Nature Portfolio policies, see our Editorial Policies and the Editorial Policy Checklist.

Statistics

For all statistical analyses, confirm that the following items are present in the figure legend, table legend, main text, or Methods section.

n/a	Confirmed	
☐	☒	The exact sample size (*n*) for each experimental group/condition, given as a discrete number and unit of measurement
☒	☐	A statement on whether measurements were taken from distinct samples or whether the same sample was measured repeatedly
☒	☐	The statistical test(s) used AND whether they are one- or two-sided<br>*Only common tests should be described solely by name; describe more complex techniques in the Methods section.*
☐	☒	A description of all covariates tested
☒	☐	A description of any assumptions or corrections, such as tests of normality and adjustment for multiple comparisons
☐	☒	A full description of the statistical parameters including central tendency (e.g. means) or other basic estimates (e.g. regression coefficient) AND variation (e.g. standard deviation) or associated estimates of uncertainty (e.g. confidence intervals)
☒	☐	For null hypothesis testing, the test statistic (e.g. *F*, *t*, *r*) with confidence intervals, effect sizes, degrees of freedom and *P* value noted<br>*Give P values as exact values whenever suitable.*
☒	☐	For Bayesian analysis, information on the choice of priors and Markov chain Monte Carlo settings
☒	☐	For hierarchical and complex designs, identification of the appropriate level for tests and full reporting of outcomes
☒	☐	Estimates of effect sizes (e.g. Cohen's *d*, Pearson's *r*), indicating how they were calculated

Our web collection on statistics for biologists contains articles on many of the points above.

Software and code

Policy information about availability of computer code

Data collection	The data for this study was provided by the Israeli Ministry of Health. No special software was used
Data analysis	Data analysis was done in R (version 4.1.2), using standard data analysis packages (dplyr version 2.1.1) and regression analysis

For manuscripts utilizing custom algorithms or software that are central to the research but not yet described in published literature, software must be made available to editors and reviewers. We strongly encourage code deposition in a community repository (e.g. GitHub). See the Nature Portfolio guidelines for submitting code & software for further information.

Data

Policy information about availability of data

All manuscripts must include a data availability statement. This statement should provide the following information, where applicable:

- Accession codes, unique identifiers, or web links for publicly available datasets
- A description of any restrictions on data availability
- For clinical datasets or third party data, please ensure that the statement adheres to our policy

The individual-level data used in this study are sensitive and cannot be publicly shared.

Field-specific reporting

Please select the one below that is the best fit for your research. If you are not sure, read the appropriate sections before making your selection.

☒ Life sciences ☐ Behavioural & social sciences ☐ Ecological, evolutionary & environmental sciences

For a reference copy of the document with all sections, see nature.com/documents/nr-reporting-summary-flat.pdf

Life sciences study design

All studies must disclose on these points even when the disclosure is negative.

Sample size	This is an observational quasi-experimental study based on data on all population in Israel in the studied age groups.
Data exclusions	We used a quasi-experimental design, utilizing the changes made to the vaccine eligibility age cutoffs to estimate the effectiveness of a booster dose to that of a "fresh" 2-dose vaccine. The study population included persons who were between the ages of 12-14 (51.9% female, 48.1% male) or 16-18 (50.6% female, 49.4% male) starting January 1st, had no documented positive PCR result prior to the study period, had not stayed abroad during the whole study period, and had not been vaccinated with a vaccine different from BNT162b2 before the end of the study period. We did not include the 15-year-old group since the data included the age of individuals in one-year groups (based on their age on January 1st, 2021), and the 15-year-old group thus included individuals who were eligible to vaccinate at different times.
Replication	NA (This is an observational quasi-experimental study based on data on all population in Israel in the studied age groups.)
Randomization	NA. Quasi-experimental study where individuals belonged to specific study groups based on their age and vaccination status
Blinding	NA. People chose whether to receive the vaccine (not a randomized trial)

Reporting for specific materials, systems and methods

We require information from authors about some types of materials, experimental systems and methods used in many studies. Here, indicate whether each material, system or method listed is relevant to your study. If you are not sure if a list item applies to your research, read the appropriate section before selecting a response.

Materials & experimental systems

n/a	Involved in the study	
☒	☐	Antibodies
☒	☐	Eukaryotic cell lines
☒	☐	Palaeontology and archaeology
☒	☐	Animals and other organisms
☐	☒	Human research participants
☒	☐	Clinical data
☒	☐	Dual use research of concern

Methods

n/a	Involved in the study	
☒	☐	ChIP-seq
☒	☐	Flow cytometry
☒	☐	MRI-based neuroimaging

Human research participants

Policy information about studies involving human research participants

Population characteristics	he study population included persons who were between the ages of 12-14 (51.9% female, 48.1% male) or 16-18 (50.6% female, 49.4% male) starting January 1st, had no documented positive PCR result prior to the study period, had not stayed abroad during the whole study period, and had not been vaccinated with a vaccine different from BNT162b2 before the end of the study period. We did not include the 15-year-old group since the data included the age of individuals in one-year groups (based on their age on January 1st, 2021), and the 15-year-old group thus included individuals who were eligible to vaccinate at different times.
Recruitment	NA. The data includes information about all individuals in the relevant age groups as provided by the Ministry of Health.
Ethics oversight	The study was approved by the Institutional Review Board of the Sheba Medical Center. Helsinki approval number: SMC-8228-21

Note that full information on the approval of the study protocol must also be provided in the manuscript.
